# Biosensor-aided isolation of anaerobic arsenic-methylating bacteria from soil

**DOI:** 10.1093/ismeco/ycaf081

**Published:** 2025-05-09

**Authors:** Hugo Sallet, Luna Kaiser, Matteo Titus, Marion Calvo, Nicolas Jacquemin, Karin Lederballe Meibom, Rizlan Bernier-Latmani

**Affiliations:** Ecole Polytechnique Federale de Lausanne (EPFL), Environmental Microbiology Laboratory, Lausanne, CH-1015, Switzerland; Ecole Polytechnique Federale de Lausanne (EPFL), Environmental Microbiology Laboratory, Lausanne, CH-1015, Switzerland; Ecole Polytechnique Federale de Lausanne (EPFL), Environmental Microbiology Laboratory, Lausanne, CH-1015, Switzerland; Ecole Polytechnique Federale de Lausanne (EPFL), Environmental Microbiology Laboratory, Lausanne, CH-1015, Switzerland; Ecole Polytechnique Federale de Lausanne (EPFL), Environmental Microbiology Laboratory, Lausanne, CH-1015, Switzerland; Ecole Polytechnique Federale de Lausanne (EPFL), Environmental Microbiology Laboratory, Lausanne, CH-1015, Switzerland; Ecole Polytechnique Federale de Lausanne (EPFL), Environmental Microbiology Laboratory, Lausanne, CH-1015, Switzerland

**Keywords:** arsenic methylation, paddy soil, biosensor, isolation, anaerobic bacteria, hydrogel capsules, FACS, arsenate reduction

## Abstract

Microbial methylation of arsenic impacts both the toxicity and fate of this environmental contaminant and is an important component of its biogeochemical cycle. This transformation occurs in flooded paddy fields where soil microorganisms can produce dimethylated arsenic, which causes the straighthead disease in rice. The responsible anaerobic microorganisms have remained elusive because their isolation is laborious, especially as the active methylators cannot be rapidly screened. Here, we introduce a novel approach to specifically target these microorganisms. This approach is based on a high-throughput isolation technique involving microfluidic encapsulation, fluorescence-activated cell sorting, and biosensor-aided screening of microbial function. Using this method, we isolated two arsenic-methylating anaerobes from a paddy soil. This approach has the potential to rapidly obtain novel isolates. For instance, we show that one isolate actively methylates arsenate (As^V^), a previously unknown phenotype in anaerobes.

## Introduction

Arsenic (As) is a toxic metalloid that is ubiquitous in the environment. Though primarily confined to rocks (e.g. in sulfide minerals), its presence in the biosphere originates from geogenic processes (e.g. weathering, volcanic emissions) and anthropogenic activities (e.g. mining, agriculture) [1, 2]. In soil and groundwater, arsenic occurs as the inorganic compounds arsenite (As^III^) and arsenate (As^V^), which predominate under reducing and oxidizing conditions, respectively. Some microorganisms can enzymatically convert As^III^ to mono-, di-, and trimethylated compounds (MMAs^III^, DMAs^III^, and TMAs^III^, respectively). This biotransformation, referred to as arsenic methylation, alters the fate and toxicity of the contaminant, thus playing a pivotal environmental role.

In paddy fields, the rice plant (*Oryza sativa*) inadvertently takes up arsenic, both As^III^ (naturally present in the porewater) and its methylated products MMAs^III^ and DMAs^III^ (produced by soil microorganisms), which are further translocated to its grains, i.e. entering the food chain [3]. MMAs^III^ and DMAs^III^ are considered to be more cytotoxic and genotoxic to humans than As^III^ [4–7], and can be further thiolated (abiotically) to form more toxic compounds which also accumulate in rice grains [8, 9]. Additionally, dimethylarsenate (DMAs^V^), the pentavalent derivative of DMAs^III^, is the causative agent of straighthead, a disease inducing the sterility of rice crops [3, 10]. Arsenic methylation is thus a threat to both food safety and security, especially as rice is a staple food for more than half of the world’s population [11–13]. Additionally, the final step of the microbial transformation is key to the arsenic biogeochemical cycle since its product (TMAs^III^) is volatile and thus released into the atmosphere [14]. Although arsenic volatilization via microbial methylation is often negligible [15–17], this route has been proposed as a bioremediation strategy to remove the metalloid from polluted fields [18–20], especially as TMAs^III^ is relatively non-toxic [21].

The enzymatic mechanism of arsenic methylation has been extensively studied [22–26]. The reaction occurs in the cytoplasm and is catalyzed by the enzyme arsenite *S*-adenosylmethionine methyltransferase (ArsM), which transfers methyl groups from *S-*adenosylmethionine (SAM) to the bound substrate (e.g. As^III^) [22]. ArsM is generally encoded in an *ars* operon along with genes involved in arsenic resistance, and its expression is regulated by the transcriptional repressor ArsR [27]. *arsM* genes have been used as a proxy to assess the abundance and diversity of arsenic-methylating microorganisms in paddy soils [16, 28, 29], revealing their widespread occurrence across phylogenetically diverse taxa [30, 31].

However, the biotic and abiotic factors driving arsenic methylation in *arsM*-bearing microorganisms are poorly understood, as substantial variations in methylation efficiencies have been reported in pure cultures [32]. It has been found that a greater intracellular accumulation of As^III^ increases methylation activity [20, 24, 32]. Additionally, in paddy soils, the transformation is enhanced upon flooding [16, 33, 34] because of the solubilization of As^III^ in porewater, but possibly also because of the increased activity of anaerobes, which dominate under these conditions [34]. Yet anaerobes have been found to be less efficient methylators than aerobes in pure culture [29, 32]. Since stronger methylation activity is generally observed in anaerobic enrichment cultures than in anaerobic pure cultures [29, 35–37], interspecies interactions may partly mediate this process in flooded soils.

Additionally, the ecological function of arsenic methylation remains unclear. The transformation was originally considered a detoxification mechanism because heterologous expression of *arsM* genes in *Escherichia coli* increased resistance to As^III^ [22, 26, 38–40]. This hypothesis is widely accepted for aerobic methylators, where methylated products are rapidly oxidized to their pentavalent forms, which are less toxic than As^III^ [26, 41]. In contrast, in anaerobic methylators, the trivalent products are preserved due to the absence of oxygen, and they are more toxic than As^III^. There is a growing body of evidence supporting that this phenotype may confer a competitive advantage by inhibiting microbial competitors [42–45]. The *arsM* gene is thought to have emerged before the Great Oxidation Event [46, 47], i.e. at a time when oxygen was absent or scarce, and was conserved throughout the long evolutionary history of microbial life. Thus, it must improve the fitness of anaerobes, yet the nature of this benefit remains ambiguous.

Such complex biological questions cannot be rigorously addressed with soil microcosms or enrichment cultures because of the large number of abiotic and biotic biases hampering hypothesis testing. In contrast, experiments involving pure cultures of environmentally-relevant isolates, or synthetic communities assembled from these, may yield more insightful and reproducible results. Thus, there is a need to isolate arsenic-methylating anaerobes from paddy soils. Recent findings have revealed a large diversity in the structure and function of ArsM enzymes [24, 26, 35, 46], as well as novel *arsM* expression mechanisms [48]. This strengthens the importance of isolating novel strains to expand our knowledge of arsenic methylation.

However, anaerobic arsenic-methylating isolates remain very limited in number and diversity. Isolating such organisms from paddy soil is challenging. The traditional isolation technique (colony picking on agar) is ill-suited for environmental anaerobes, many of which grow slowly and require stringent conditions. Alternatives (dilution-to-extinction, Hungate roll-tubes [49]) are cumbersome and low-throughput, thus inefficient for isolating organisms with rare phenotypes in complex microbiomes such as soil. Furthermore, there is no means to systematically screen for microorganisms that methylate arsenic, and since the phenotype is not related to a particular taxonomic group or metabolism, these cannot be enriched in culture.

Due to these challenges, arsenic-methylating anaerobes have been isolated with untargeted approaches [35, 36, 40], which consists of generating large numbers of isolates, without prior selection or screening, and individually testing these in pure culture for methylation activity. To do so, analytical systems for arsenic speciation measurement, such as high-performance liquid chromatography-inductively coupled plasma mass spectrometry (HPLC-ICP-MS), are used. In some cases, arsenic methylation activity was discovered fortuitously in microorganisms originally isolated to study unrelated transformations [36, 50]. In contrast, one arsenic-methylating anaerobe (*Paraclostridium bifermentans* EML) was isolated with a targeted approach based on an extensive meta-omics analysis where individual members of a soil-derived community were profiled so as to identify the active methylators (i.e. those that express *arsM* and produce ArsM) [37]. Targeted approaches yield much higher success rates in obtaining individual organisms with the desired phenotype. Nonetheless, meta-omics methods are costly, time-consuming, and ill-suited for routine isolation of specific microbial functions.

The goal of this study is to introduce a novel targeted approach that facilitates the isolation of arsenic-methylating anaerobes. The method is based on two steps: (i) high-throughput generation of isolates by microfluidic single-cell encapsulation and fluorescence-activated cell sorting (FACS) and (ii) rapid screening of isolates with a whole-cell bacterial biosensor responsive to methylated arsenic. We used this targeted approach to isolate two anaerobes from a paddy soil that actively methylate arsenic. In addition to methylating As^III^, one isolate (*Paraclostridium dentum* IIB4) exhibited the ability to actively methylate As^V^, a phenotype not previously explored in anaerobic microorganisms.

## Materials and methods

### Arsenic speciation analysis

Arsenic speciation was measured by reversed-phase HPLC with a C18 column (part 00G-4053-E0, Phenomenex, Torrance, CA, USA) coupled to an 8900 Triple Quadrupole ICP-MS (Agilent Technologies, Santa Clara, CA, USA). The eluent (5 mM tetrabutylammonium hydroxide, 2 mM malonic acid, 5% methanol, pH = 5.83) was pumped through the column at a flow rate of 1 ml/min. Prior to analysis, samples were filtered (0.22 μm) and diluted in 1% HNO_3_. When assessing the dynamics of arsenic transformations in pure strains, samples were additionally oxidized in 10% H_2_O_2_. For intracellular As, pellets of 1 ml of culture (8000 g, 5 min) were washed once in phosphate-buffered saline (PBS), resuspended in 1 ml of lysis buffer (STET (Sodium chloride, Tris-Cl, EDTA, Triton X-100), 10 mg/ml lysozyme) and incubated for 1 h at 37°C. The resulting lysate was centrifuged (12 000 g, 5 min) to remove cell debris. The list of As chemical species and their abbreviations is provided in Table S1.

### Biosensor design

Biosensor strains consisted of *E. coli* MG1655 Δ*arsRBC* [51] with a plasmid containing the biosensor elements. To construct the plasmids, DNA fragments were designed containing two elements: (i) the *arsR* gene from *Noviherbaspirillum denitrificans* HC18 (locus tag EGT07_24170, Genbank no. RZI40430) or *Shewanella putrefaciens* 200 (locus tag Sput200_1227) preceded by a ribosome binding site and a constitutive promoter (AA-promoter [52]) and (ii) the *arsRM* promoter from *N. denitrificans* (containing the 147 bp upstream of the start and the first 48 bp of the *arsR* gene) or the *arsP* promoter from *S. putrefaciens* (encompassing the 222 bp upstream of the start of *arsP*; Sput200_1226). The *arsRM* and *arsP* promoters are regulated by their respective ArsR proteins and drive *gfp* expression in the plasmids. The *arsR* genes were codon optimized for expression in *E. coli* and furthermore, restriction sites were introduced to facilitate cloning (*Xba*I and *Eco*RI sites at extremities) and to allow deletion of *arsR* (*Bam*HI site before and after *arsR* gene). The designed fragments (Nd-ars-gBlock, Sp-ars-gBlock) were synthesized by Integrated DNA Technologies (Coralville, IA, USA). The sequences are reported in Text S3. The gBlocks were ligated into pCR-Blunt (Thermo Fisher Scientific), the plasmids purified and digested by XbaI and EcoRI to liberate the gBlocks. Plasmid pAAK12 (containing an As^III^ biosensor based on *arsR* from *E. coli* [51]) was similarly digested with *Xba*I and *Eco*RI and the fragment containing the *ars*-promoter and *arsR* gene, including its promoter, was replaced with the Nd-ars-gBlock or Sp-ars-gBlock, resulting in plasmids pAA-Nd-arsBS and pAA-Sp-arsBS (Fig. S1). Two control plasmids without *arsR* were produced by excising the *arsR* gene by *Bam*HI digestion of pAA-Nd-arsBS and pAA-Sp-arsBS, followed by ligation, resulting in plasmids pAA-Nd-arsKO and pAA-Sp-arsKO. All constructs were verified by Sanger sequencing (Microsynth, Balgach, Switzerland) using a plasmid-specific primer (5’-CTGCCAGGAATTGGGGATC-3′) before introduction into *E. coli* Δ*arsRBC*. For simplicity, the resulting strains are hereafter referred to as Nd-biosensor and Sp-biosensor.

**Figure 1:**
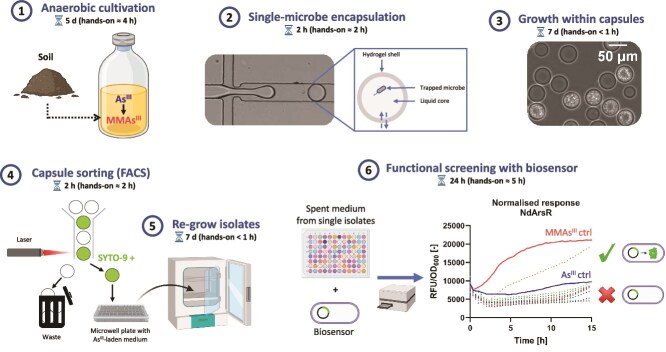
Workflow of the isolation protocol. The total duration of each step is specified, as well as the estimated hands-on time. Part of the workflow is adapted from Sallet *et al.* [53].

### Biosensor assays in the presence of various arsenic compounds

Biosensor strains were cultured in ¼ TSB medium containing 50 μg/ml kanamycin and amended with varying amounts of arsenic compounds (2–50 μM As^III^, 0.05–1 μM MMAs^III^, 0.1–5 μM DMAs^III^, 0.1–2 μM MMAs^V^, 0.2–7 μM DMAs^V^) to assess their response to these compounds during growth. Cultures were prepared in 96-well plates. Mineral oil (40 μl) was added on top of each microwell to limit oxygen diffusion and thus prevent the oxidation of trivalent arsenic. Incubation was performed at 37°C in a microplate reader (Synergy MX, BioTek Instruments, Winooski, VT, USA), where both fluorescence (λ_exc_: 479 nm, λ_em_: 520 nm) and optical density (OD_600_) were measured continuously over a 15-h period.

### Assessment of biosensor response to As^III^ biomethylation

Next, we tested whether the biosensors could detect biological As methylation. *P. bifermentans* EML [37] was cultivated at 30°C in ¼ TSB medium (amended with As^III^) for ~24 h. Initial As^III^ concentrations were 5 μM and 9 μM for the experiment with Nd-biosensor and Sp-biosensor, respectively. At various incubation times, an aliquot of the culture was collected and filtered (0.22 μm), and the resulting spent medium was stored anoxically. Biosensor strains were subsequently grown in the spent media (i.e. the samples collected at the different times) amended with kanamycin (50 μg/ml). The biosensor cultures were prepared in 96-well plates (each microwell was topped with 40 μl mineral oil) and incubated at 37°C in a microplate reader for 15 h over which fluorescence and OD_600_ were measured.

### Isolation of arsenic-methylating soil anaerobes

Microorganisms were extracted from a Swiss flooded paddy soil (soil A_AH_ in Sallet *et al.* [53]) as described previously [53], and cultured anaerobically in various media (TSB, DSMZ 141c, 2x YTG, Postgate and mEA, see Text S2 for details) amended with As^III^ (10 μM) at 30°C for 5 days. Only the TSB and DSMZ 141c cultures were subsequently used in the isolation experiment, following the procedure described in Sallet *et al.* [53] and the workflow represented diagrammatically in Fig. 1. Briefly, single microbial cells were trapped in semi-permeable hydrogel capsules and incubated (30°C, 1 week) in medium to allow cells to grow within; the biomass was then fluorescently stained (2 μM SYTO-9) and high-SYTO-9 capsules (i.e. those with high biomass) were sorted (FACS) and distributed individually in microwell plates filled with As^III^-laden (25 μM) medium. The isolates (i.e. derived from individual capsules) were further cultured (30°C) anoxically (H_2_/CO_2_ (80/20 v/v) headspace) in the same medium (TSB or DSMZ 141c). After one week, the supernatant (i.e. spent medium) of each well showing visible biomass was amended (10% v/v) to freshly started cultures of Nd-biosensor (¼ TSB, 50 μg/ml kanamycin, 40 μl mineral oil) incubated for 15 h in a microplate (similar to above). Positively screened isolates, i.e. those whose spent medium induced a stronger biosensor response than the control (i.e. 2.5 μM As^III^), were further cultured for 5 days in the presence of As^III^ (25 μM) for arsenic speciation measurement.

## Results

### Detection of arsenic methylation activity with whole-cell bacterial biosensors

We recently developed a high-throughput isolation method [53] (Fig. 1), which we sought to employ for the isolation of anaerobes capable of arsenic methylation. Since this phenotype is rare among microorganisms, we constructed an *E. coli* biosensor that could specifically screen for functional isolates.

Similarly to previous constructs, the sensing mechanism is based on the arsenic-sensitive transcriptional repressor ArsR [41, 48, 54]. In the absence of arsenic, ArsR inhibits gene expression by binding to the promoter. When present, arsenic binds to ArsR, which induces its dissociation from DNA, allowing transcription to occur. Signal transduction is ensured by placing a reporter gene (*gfp*) under the control of a promoter repressed by ArsR. Since ArsR generally binds As^III^, we leveraged two non-canonical ArsR proteins, previously reported to specifically respond to MMAs^III^, from *N. denitrificans* HC18 and *S. putrefaciens* 200 [48, 54], to construct Nd-biosensor and Sp-biosensor strains, respectively.

**Figure 2:**
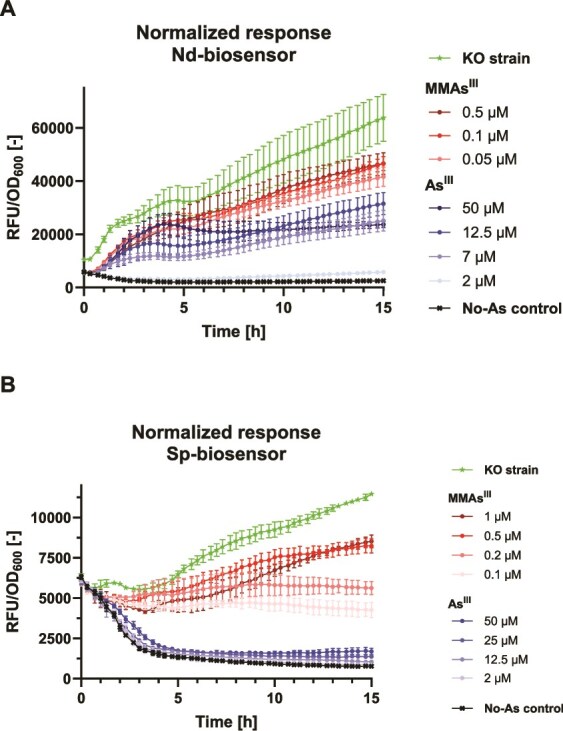
Response of biosensor strains to As^III^ and MMAs^III^. (A) Normalized fluorescence response monitored in cultures of (A) Nd-biosensor and (B) Sp-biosensor amended with various amounts of As^III^ and MMAs^III^. Shown are means of four replicates, with error bars indicating standard deviation. RFU, relative fluorescence unit. OD_600_, optical density read at 600-nm wavelength.

Both biosensor strains showed a much stronger response to MMAs^III^ than to As^III^ (Fig. 2). In the Nd-biosensor cultures (Fig. 2A), the concentrations of 0.1 μM and 0.5 μM MMAs^III^ induced the strongest signal after 15 h, which was 72% of that measured in the positive control (Nd-biosensor-KO, containing a plasmid with no repressor). The strain responded to a lower extent to As^III^, where the strongest signal was obtained with 12.5 μM As^III^ (47% of that of Nd-biosensor-KO) after 15 h. The lowest concentration of MMAs^III^ tested (0.05 μM) still resulted in a 32% and 75% stronger signal than that induced by 12.5 μM and 50 μM As^III^, respectively, after 15 h.

In the Sp-biosensor cultures, the response to As^III^ was very low, with a maximum signal obtained with 50 μM As^III^ (highest concentration tested) after 15 h, reaching 9% of that of the KO strain (Fig. 2B). In contrast, MMAs^III^ induced a strong response, peaking with 1 μM MMAs^III^ (73% of that of Sp-biosensor-KO) after 15 h. Further, a concentration of 0.1 μM MMAs^III^ yielded a 151% stronger response than 50 μM As^III^ after 15 h.

Nd-biosensor cells were found to yield much stronger fluorescence signal than those of Sp-biosensor (5-fold difference between the two KO strains after 15 h), regardless of the arsenic treatment (Fig. 2). Also, Nd-biosensor – but not Sp-biosensor – was found to respond to DMAs^III^ (Fig. S2). In both strains, however, no response was induced by MMAs^V^ (Fig. S3) or DMAs^V^ (Fig. S4). The biosensors can also be used on agar plates (Fig. S5, Text S4).

**Figure 3:**
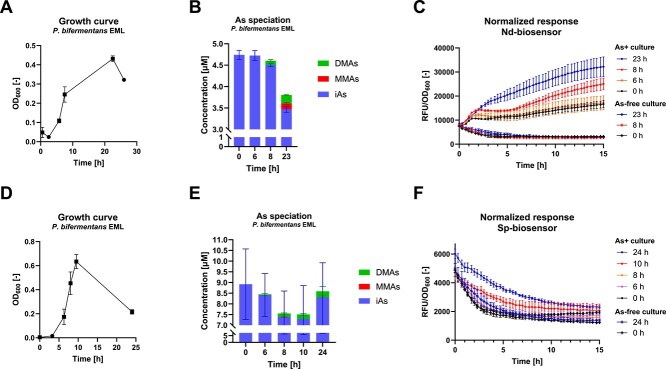
Detection of microbial As^III^ methylation by biosensor strains. (A) Growth and (B) arsenic speciation in cultures of *P. bifermentans* EML in ¼ TSB medium amended with 5 μM As^III^. (C) Normalized fluorescence response of Nd-biosensor grown in the spent medium of the strain EML cultures. The selected time points (0 h, 6 h, 8 h, 23 h) in (B) and (C) correspond to those labeled as squares in (A). (D) Growth and (E) arsenic speciation in cultures of *P. bifermentans* EML in ¼ TSB medium amended with 9 μM As^III^. (F) Normalized fluorescence response of Sp-biosensor grown in the spent medium of the strain EML cultures. The selected time points (0 h, 6 h, 8 h, 10 h, 24 h) in (E) and (F) correspond to those labeled as squares in (A). Graphs show mean and standard deviation, with duplicates for (A), (B), (D), and (E), and six replicates for (C) and (F). iAs, inorganic arsenic, MMAs, monomethylated arsenic, DMAs, dimethylated arsenic.

Further, we tested whether microbial methylation could be detected by the biosensors using a pure culture of *P. bifermentans* EML, a soil-derived arsenic methylating anaerobe [37]. In the experiment conducted to test the Nd-biosensor, arsenic methylation by strain EML was confirmed during the exponential (and possibly the stationary) phase (Fig. 3A-B), by the analytical detection of methylated products in the culture supernatant after 8 h (0.1 μM DMAs) and 23 h (0.2 μM DMAs and 0.1 μM MMAs). The biosensor fluorescence (at 15 h growth) increased by 48% and 91% after addition of the 8-h and 23-h medium supernatant, respectively (Fig. 3C). No response was induced by the supernatant from the strain EML cultures that were not amended with As^III^ (Fig. 3C).

In the experiment with the Sp-biosensor, DMAs was also detected in the supernatant of strain EML cultures after similar growth stages (Fig. 3D-E), i.e. at 6 h (0.04 μM), 8 h (0.16 μM), 10 h (0.21 μM) and 24 h (0.28 μM) time points. However, no MMAs was detected in this experiment (Fig. 3E). The biosensor response was only clear for the supernatant supplements from the 10-h and 24-h time points (Fig. 3F), with an increase in fluorescence of 39% and 78% respectively, after 7 h of incubation of the Sp-biosensor strain.

### High-throughput isolation of arsenic-methylating anaerobes

We set out to leverage the biosensors to facilitate the isolation of arsenic-methylating anaerobes. The Nd-biosensor was selected for the isolation experiment since it showed a higher sensitivity to MMAs^III^, also responded to DMAs^III^, and produced a stronger fluorescent signal.

**Figure 4:**
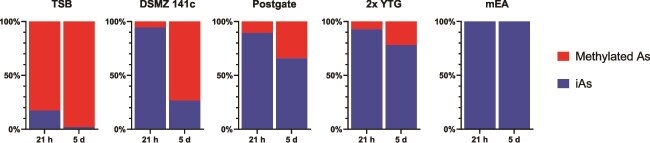
Arsenic methylation in paddy soil-derived microbial cultures grown in various media. Proportion of methylated arsenic produced in the culture medium of paddy soil-derived enrichments after 21 h and 5 days of incubation. iAs, inorganic arsenic.

Methylation took place in all paddy soil-derived anaerobic cultures, prepared in various media amended with As^III^, except in mEA medium (Fig. 4). MMAs was the main product (data not shown). Methylation was particularly efficient in the TSB and 141c cultures, where 98% and 73% of arsenic was methylated after 5 days, respectively.

Therefore, these two enrichment cultures were selected for the isolation experiment. The approach employed – combining microfluidic single-cell encapsulation and FACS—resulted in the cultivation of 298 isolates in As^III^-amended medium. The spent medium of each isolate was screened with the Nd-biosensor strain. While the spent medium of most isolates induced a low biosensor response (i.e. similar signal to that expected from the As^III^ added initially in the medium), some induced a much stronger response (Fig. 1). These corresponding isolates (56 total, all those that triggered a response slightly above that of the 2.5 μM As^III^ control) were further cultured to check their function by arsenic speciation analysis.

Methylation was confirmed in two isolates, both derived from 141c cultures (Table 1). The main product was MMAs (data not shown). Five more isolates (three derived from 141c and two from TSB cultures) also showed methylation activity but were not pure. Interestingly, another isolate (derived from the 141c culture) produced an unknown arsenical, resulting in an unidentified peak in the chromatogram. In the remaining isolates, methylated arsenic was either not detected or detected at low levels (near detection limit), so those were not further investigated.

**Table 1:** List of isolates obtained from the paddy soil.

**Label**	**Taxonomy**	**Enrichment culture**	**Methylated As [%]** ^a^
IIB7	*T. petrolearius* ^b^	DSMZ 141c	21.1
IIB4	*P. dentum* ^b^	DSMZ 141c	28.4
IC2	*P. bifermentans* ^c^ (mixed culture)	TSB	18.4
IA5	*P. bifermentans* ^c^ (mixed culture)	TSB	15.5
IID7	Unknown (mixed culture)	DSMZ 141c	14.9
VH12	Unknown (mixed culture)	DSMZ 141c	16.9
IIIC11	Unknown (mixed culture)	DSMZ 141c	4.5
IVA1	Unknown (mixed culture)	DSMZ 141c	0 (11.6% of unknown arsenical)

a Measured in the medium after incubation for 5 days.

b Taxonomy determined from whole-genome sequences (PacBio sequencing).

c Taxonomy determined from 16S rRNA gene amplicon sequences (Sanger sequencing).

### As^III^ methylation dynamics in soil isolates

**Figure 5:**
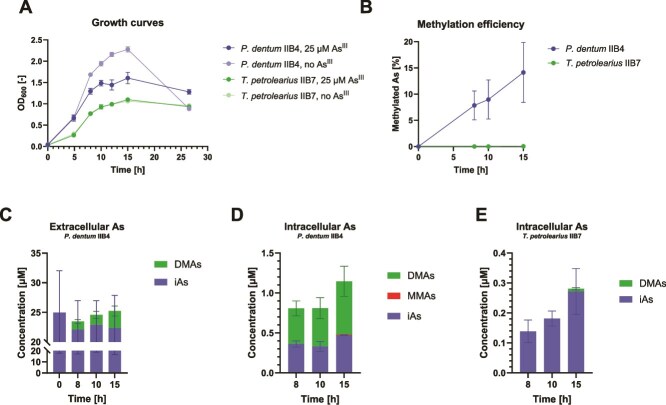
As^III^ methylation dynamics in pure cultures of *P. dentum* IIB4 and *T. petrolearius* IIB7. (A) Growth profile and (B) methylation efficiency (proportion of methylated arsenic) in pure cultures of *P. dentum* IIB4 and *T. petrolearius* IIB7. Arsenic speciation in (C) the culture medium (D) and inside the cells of strain IIB4, or (E) inside the cells of strain IIB7. iAs, inorganic arsenic. MMAs, monomethylated arsenic. DMAs, dimethylated arsenic.

The methylation activity of the two pure isolates, *P. dentum* IIB4 and *Terrisporobacter petrolearius* IIB7, was further investigated in pure culture. Both strains could grow in the presence of As^III^ (25 μM), but the growth of strain IIB4 was negatively impacted by the arsenical, while strain IIB7 was seemingly insensitive to it (Fig. 5A). Strain IIB4 was able to methylate 14% of As^III^ in 15 h (Fig. 5B). Methylation was observed already after 8 h of incubation, i.e. during the exponential phase (Fig. 5A-B). DMAs was produced in the medium (Fig. 5C), and represented more than half of the intracellular arsenic (Fig. 5D). Although not measured in the medium, trace levels of MMAs (0.8%) were detected inside IIB4 cells. In contrast, no methylation was observed in the culture of strain IIB7 (Fig. 5B, S6). However, DMAs (3.1%) was detected in the biomass after 15 h (Fig. 5E), i.e. during the stationary phase (Fig. 5A). When the experiment was repeated by growing strain IIB7 over a longer incubation period (4 days), methylated products (3% DMAs, 24% TMAs) were measured in the medium (Table 2).

**Table 2:** Methylation of *T. petrolearius* IIB7 after 4-day incubation (TSB, 30 μM As^III^).

	**iAs**	**MMAs**	**DMAs**	**TMAs**
**Concentration** ^a^ **(μM)**	22.81 ± 1.47	0.00 ± 0.00	0.94 ± 0.06	7.58 ± 0.25

a Mean ± standard deviation (three replicates) are shown.

We also tested the methylation activity in another strain, *T. petrolearius* 6C8, which we had isolated from the same soil [53], since it belongs to the same species as strain IIB7. Stain 6C8 methylated As^III^ during the exponential phase and stationary phase (Fig. S7A-B), releasing DMAs and TMAs in the medium (8% and 2% after 30 h, respectively) (Fig. S7C), while MMAs and DMAs were found in the biomass (1% and 18% after 30 h, respectively) (Fig. S7D).

**Figure 6:**
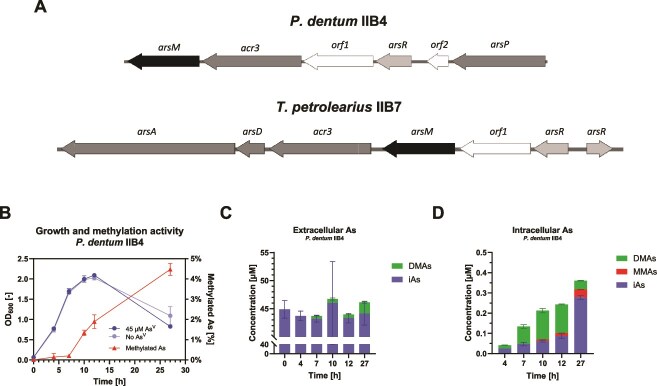
Methylation of as^V^ by *P. dentum* IIB4. (A) Diagram of the *ars* operon in *P. dentum* IIB4 and *T. petrolearius* IIB7. A description of the genes and their product is as follows. *arsM*: As^III^  *S*-adenosylmethionine methyltransferase (ArsM), *orf1*: Putative As^V^ reductase (Orf1), *acr3*: As^III^ permease (Acr3), *arsR*: Arsenic-responsive transcriptional repressor (ArsR), *arsP*: Putative MMAs^III^ permease (ArsP), *arsD:* As^III^ metallochaperone, *arsA:* Efflux pump-powering ATPase, *orf2*: Thioredoxin family protein. (B) Growth and As^V^ methylation activity of *P. dentum* IIB4 in pure culture, and arsenic speciation dynamics (C) in the culture medium and (D) inside the cells. iAs, inorganic arsenic. MMAs, monomethylated arsenic. DMAs, dimethylated arsenic.

For each of these strains, an *ars* operon comprising an *arsM* gene was found either on a plasmid (strain IIB4) or on the chromosome (strains IIB7 and 6C8) (Figs 6A and S8).

### *Paraclostridium dentum* IIB4 produces methylated arsenicals from As^V^

Interestingly, the three strains were also found to harbor a metallophosphoesterase-encoding gene (*orf1*) in their *ars* operon (Figs 6A and  S8). Orf1 was found to be ~40% identical and ~60% similar to Car1, an As^V^ reductase recently identified in *Rufibacter tibetensis* [55] and presents the cysteine residues (Cys74, Cys76) essential to the reductase activity in this organism. Strain IIB4 was thus grown in the presence of As^V^ to test for both reduction and methylation activity. Methylation was observed over the whole growth period (Fig. 6B) and resulted in the release of DMAs (4% after 27 h) in the medium (Fig. 6C). Methylated products were detected in the biomass after only 4 h (Fig. 6D). Apart from DMAs, MMAs was found inside the cells, particularly at later times (11% after 27 h) (Fig. 6D).

## Discussion

Although recent evidence points to the crucial role of anaerobes in arsenic methylation in paddy soils [34, 35], their isolation has remained challenging due to the technical difficulty to culture individual anaerobic strains and the absence of a method to screen for this phenotype.

Until now, arsenic-methylating anaerobes have been isolated by generating large numbers of soil-derived isolates (with dilution-to-extinction or roll-tube methods) and subsequently testing each of these for methylation activity by arsenic speciation analysis. Such untargeted approaches are tedious, time-consuming, and require the intensive usage of complex analytical tools (e.g. HPLC-ICP-MS) that are not always available to researchers. In contrast, we have previously employed meta-omics tools to identify microorganisms showing arsenic methylation activity in a paddy soil-derived community [37], which has guided the isolation of the anaerobic methylator *P*. *bifermentans* EML. This approach was finely targeted and thus had a high chance of success, but it required significant time and resources. Thus, it is not a sustainable strategy for the isolation of specific functional groups of microbes.

Here, we introduce a novel, high-throughput, targeted approach that addresses this challenge, leveraging, on the one hand, a recent microfluidics-based isolation technique involving single-cell compartmentalization and FACS [53], and on the other hand, an MMAs^III^- and DMAs^III^-responsive *E. coli* biosensor for functional screening (Fig. 1).

The design of the biosensor strains was based on two non-canonical ArsR proteins previously reported [48, 54]. In the original studies, biosensors were constructed in *E. coli* to test their ability to detect arsenicals *in vitro* (response measured after a single time point). Here, we leveraged our biosensors to detect biological methylation and monitored their response continuously over the growth period (i.e. 15 h). Additionally, we measured a strong fluorescence response at MMAs^III^ concentrations lower than previously reported (0.5 μM), even below 0.1 μM (Fig. 2). Although based on the same ArsR proteins, our biosensors differ from previous ones in their expression system —*arsR* and *gfp* being located on a single plasmid and *arsR* being expressed constitutively — and in the host strain (MG1655 Δ*arsRBC*). These differences may explain the enhanced activity in the present biosensor strains. Such a high sensitivity to MMAs^III^ is critical for screening arsenic-methylating anaerobes, which may produce very low amounts of MMAs^III^ [32].

We found notable differences in activity between the two biosensor strains, Nd-biosensor being less specific but more sensitive to MMAs^III^ (Fig. 2). Interestingly, DMAs^III^ also induced a response in Nd-biosensor but not in Sp-biosensor (Fig. S2), which is consistent with the observation that only the former strain could detect biological methylation conducted by *P. bifermentans* EML, which yielded primarily DMAs^III^ (Fig. 3). In addition, the fluorescence signal yielded by Sp-biosensor was much lower, which is consistent with the absence of signal on agar, in contrast with Nd-biosensor (Fig. S5). These factors motivated the selection of Nd-biosensor for the isolation experiment (Fig. 1).

Biosensor-aided screening allowed us to select only a fraction of isolates (56/298) for which arsenic speciation analysis was performed. Hence, this approach considerably alleviates the usage of analytical instruments (typically, HPLC-ICP-MS), and thus the overall time of the experiment. A higher biosensor fluorescence threshold could be used to further lower the chance a false positive (i.e. ensure the screened isolates are real methylators).

There is, to our knowledge, only one study that previously reported the use of whole-cell biosensors to facilitate the isolation of specific microorganisms (in that case, agmatine producers from cheese) [56]. A major challenge in our work is that we sought to isolate anaerobes, for which traditional agar plating is ill-suited. We thus used a high-throughput isolation workflow recently developed for the isolation of anaerobes [53].

This approach was successful, as two novel arsenic-methylating anaerobes were isolated from a paddy soil within a short time (20 days overall, < 15 h hands-on time). The isolates, *P. dentum* IIB4 and *T. petrolearius* IIB7, were found to harbor an *arsM* gene in an *ars* operon (Fig. 6A), and to actively methylate As^III^ in pure culture (Fig. 5, Table 2). Additionally, a pure strain previously isolated from the same soil and belonging to the same species as strain IIB7, *T. petrolearius* 6C8, was also found to possess an *arsM* gene and to methylate As^III^ (Figs S7–S8). All three strains produced DMAs from As^III^, suggesting these are involved in inducing straighthead disease in rice plants. However, strain IIB7 exhibited much slower methylation activity (Figs 5E and  S6, Table 2). All three strains harbor genes encoding As^III^ efflux pumps, as all have *acr3*, and both IIB4 and IIB7 have a putative *arsB* located outside the operon. Additionally, strain IIB7 possesses genes encoding ArsA (an As transporter ATPase [57]) and ArsD (an As^III^ metallochaperone [58]) which may further enhance its As^III^ efflux capability (Fig. 6A), thus limiting intracellular accumulation and toxicity (Fig. 5A and E). Even between the two *T. petrolearius* strains, considerable differences were found in the *ars* operons (Figs 6A and  S8), and accordingly, in arsenic methylation efficiency (Figs 5B and  S7B). A third *T. petrolearius* strain recently isolated from paddy soil (strain TC13) was able to methylate MMAs^III^ but not As^III^ [35], which further emphasizes the importance of strain-level differences when considering methylation potential and thus, the need for the isolation of multiple arsenic methylating microorganisms.

Additionally, the strains were found to harbor a gene encoding a metallophosphoesterase (Orf1) similar to an As^V^ reductase recently characterized in *R. tibetensis* [55] (Figs 6A and  S8). Interestingly, *orf1* and *arsM* genes are located very closely in the *ars* operon and directly downstream an *arsR* gene. We thus hypothesized that both As^V^ reduction and As^III^ methylation could be performed by these strains. We confirmed this hypothesis by showing the active methylation of As^V^ by strain IIB4 (Fig. 6B-D). This phenotype had been observed previously in aerobic microorganisms [39, 59, 60]. However, in anaerobes, this has only been measured in 20-day-old cultures [38, 40], thus, the biological nature of this process is in question. Here, we show that As^V^ is converted to MMAs^III^ and DMAs^III^ by actively growing anaerobic bacteria (Fig. 6B–D). The co-expression of *arsC* and *arsM* has been reported in one aerobic bacterium (*Arsenicibacter rosenii* SM-1) harboring an *arsRMC* operon [61]. Here, strain IIB4 has an *arsR-orf1-acr3-arsM* operon, which suggests that, following As^V^ reduction (Orf1), As^III^ is either methylated (ArsM) or extruded (Acr3) (Figs 6A and  S8). The presence of a putative *arsP* gene (encoding an MMAs^III^ permease) suggests that the methylated product is also extruded. Thus, strain IIB4 should be able to take up As^V^ and release inorganic and organic trivalent arsenicals (both of higher toxicity than As^V^), which, under anoxic conditions, are not oxidized to their pentavalent counterparts (of lower toxicity than As^V^). The overall process likely serves anaerobic microbial fitness through detoxification and competition (microbial warfare) mechanisms.

The high methylation efficiency measured in the TSB enrichment compared with other cultures (e.g. Postgate) further suggests that fermentative bacteria, rather than sulfate-reducing bacteria (SRB), as highlighted in several studies [35, 62], may be the main drivers of arsenic methylation in some paddy soils (Fig. 4). However, the absence of methylation in the mEA culture, which contains TSB in its medium composition, indicates that the methylators were inhibited by other components in the medium, e.g. nitrate or Fe^III^.

Nevertheless, the methylation efficiency of the isolates in pure cultures (Figs 5, 6, and  S7) remains low compared with that of soil-derived enrichments (Fig. 4). Interspecies interactions may thus be involved in driving methylation activity. Alternatively, the highly active anaerobic arsenic methylators may still be missing, either because they were unable to grow in culture—possibly because specific requirements (e.g. syntrophy) were not fulfilled—or because their abundance was substantially lower than that of other microorganisms. Future efforts could thus focus on targeting consortia instead of pure strains. This can be done by increasing the number of cells initially encapsulated.

The present biosensors could also aid in the isolation of other arsenic-transforming microorganisms, e.g. those that potentially reduce pentavalent methylarsenicals or demethylate MMAs^III^. In a broader scope, the approach described herein paves the way for cultivation-oriented applications that complement meta-omics approaches, which may target anaerobes involved in crucial yet underexplored functions, such as pollutant degradation [63, 64], or antibiotic production [65], for which whole-cell biosensors have been developed.

## Supplementary Material

Supplementary_Information_ycaf081

## Data Availability

Whole genome sequencing data are accessible under the NCBI BioProject PRJNA1232622. The code used in this study is available on GitHub.
