# Supplementary material for: Biosensor-aided isolation of anaerobic arsenic-methylating bacteria from soil
Source: ISME Commun. 2025 May 9;5(1):ycaf081. doi: 10.1093/ismeco/ycaf081 (PMC12124457; doi:10.1093/ismeco/ycaf081)
Supplement: Supplementary_Information_ycaf081 [file supplementary_information_ycaf081.pdf]

Supplementary information for:

Biosensor-aided isolation of anaerobic arsenic-methylating bacteria from soil

Hugo Sallet, Luna Kaiser, Matteo Titus, Marion Calvo, Nicolas Jacquemin, Karin Lederballe Meibom, Rizlan Bernier-Latmani*

Ecole Polytechnique Federale de Lausanne (EPFL), Environmental Microbiology Laboratory, CH-1015 Lausanne

Corresponding author: Rizlan Bernier-Latmani, EPFL ENAC IIE EML CH A1 375 (Bâtiment CH), Station 6, CH-1015, Lausanne, Switzerland. Email: rizlan.bernier-latmani@epfl.ch

Text S1. Chemical reagents

The following arsenicals were used in culture amendments and analytical standards: As^{III} as sodium arsenite (Thermo Fisher Scientific, Waltham, MA, USA), As^{V} as sodium arsenate dibasic heptahydrate (Sigma-Aldrich, St. Louis, MO, USA), $MMAs^{III}$ as methyldiiodoarsine (LGC Standards, Teddington, UK), $MMAs^{V}$ as cacodylic acid (Sigma-Aldrich) and $DMAs^{V}$ as disodium methyl arsonate hydrate (Chemservice, West Chester, PA, USA). The solutions were prepared in Milli-Q water, filtered (0.22 µm) and stored anoxically in serum bottles at 4°C.

Text S2. Media preparation

All media were brought to a boil, distributed in 100-ml serum bottles (50 ml / bottle) sealed with butyl rubber stoppers and aluminium crimps. The headspace was flushed with anoxic gases (if not specified, 100% N_2) before autoclaving at 121°C for 30 min. The composition of the media used in the study is found below.

mEA medium: $NaNO_3$ (6 mM), Ferric citrate (5 mM), Yeast extract (0.01%), Cellobiose (35 mM) and 1/4 strength TSB (tryptic soy broth).

DSMZ 63 (Postgate) medium: K_2HPO_4 (0.50 g/l), NH_4Cl (1.00 g/l), Na_2SO_4 (1.00 g/l), $CaCl_2·2H_2O$ (0.10 g/l), $MgSO_4·7H_2O$ (2.00 g/l), Na-DL-lactate (2.00 g/l), Yeast extract (1.00 g/l), Sodium resazurin 0.1% w/v (0.50 ml/l), $FeSO_4·7H_2O$ (0.50 g/l), Na-thioglycolate (0.10 g/l), Ascorbic acid (0.10 g/l).

2xYTG medium: Tryptone (16 g/l), Yeast extract (10 g/l), NaCl (5 g/l), D-glucose (5 g/l), pH 6.2.

DSMZ 141c medium: KCl (0.34 g/l), $MgCl_2·6H_2O$ (4 g/l), $MgSO_4·7H_2O$ (3.45 g/l), NH_4Cl (0.25 g/l), $CaCl_2·2H_2O$ (0.14 g/l), K_2HPO_4 (0.14 g/l), NaCl (18 g/l), Modified Wolin's mineral solution (10 ml/l), $Fe(NH_4)_2(SO_4)_2·6H_2O$ 0.1% w/v (2 ml/l), Na-acetate (1 g/l), Yeast extract (2 g/l), Trypticase peptone (2 g/l), Sodium resazurin 0.1% w/v (0.5 ml/l), $NaHCO_3$ (5 g/l), Wolin's vitamin 10x solution (1 ml/l), L-Cysteine $HCl·H_2O$ (0.5 g/l), $Na_2S·9H_2O$ (0.5 g/l), Methanol (5 ml/l). CO_2/N_2 (20/80 v/v) headspace.

Wolin's 10x vitamin solution: Biotin (20 mg/l), Folic acid (20 mg/l), Pyridoxine hydrochloride (100 mg/l), Thiamine HCl (50 mg/l), Riboflavin (50 mg/l), Nicotinic acid (50 mg/l), Calcium D-(+)-Pantothenate (50 mg/l), Vitamin B_{12} (1 mg/l), p-Aminobenzoic acid (50 mg/l), (DL)-alpha-Lipoic acid (50 mg/l).

Text S3. Biosensor constructs

Sequence of Nd-ars-gBlock (5'->3'):

CCG*GAATTC*TTCATGACCGAGGCTGGCGAGGCGTTTAGCGGTAGTGGCAAGATCCATAACTTCACTATAAAACCAT
AAAAATAGTTATGTCAAGGATTTCATTTGACATTTCCAGTATTTTGGAAATATCGTATTAGTTAAGGAGGTTTTCAAT
ATGAACACCAAGGAAGCAATCTCGGCACTCGGTGCTTTGGCGCAAGAGTCGAGCTCCAATTCCGACGTCTAAGAA
ACCATTATTATTATGACATTAACCTATAAAAATAGGCGTATCACGAGGCCCTTCCGTCTTCACCTCGAGTCCCTATCA
GTGATAGAGATTGACATCCCTATCAGTGATAGAAATACTGAGCACATCAGCAGGACGCACTGACCGGATCCAAAG
GAGAGGGAAATGGATCTTGCCACGACTGCTAAGCGTTTAGCGAGCTTGGGGCACGAAAACCGTCTGGAATTATTC
CAGTTGCTGGTACAGGCAGGTCCCAACGGCATGACTATCGGAGAGCTGCAAAGCGGCTTGGACCGTCCTGCGTCC
ACGTTAGCATTCCATCTTCGTGAGTTAGTAAGCGCGGACTTAGTGACCCAAGAAAAAGAGGGGCGCATGGTTCGTT
GCCGTGCTAATTATTCGGCCCTGCACGAGGTTCTTCAGTTTGTGCAACAACGCTGTTGTCAAGGCGTAAGCATTCAA
GAGTCAGTTGCTCAGTAAGGATCCTGC*TCTAGA*

arsRM promoter (on complement), highlighted in yellow
AA-promoter, highlighted in blue
arsR, highlighted in grey
*Bam*HI sites are underlined, *Xba*I and *Eco*RI sites are in italics

Sequence of Sp-ars-gBlock (5'->3'):

CG*GAATTC*TTTTACCTCTGTGGTATTTATATTTCGAATATTCCAGAATAATAGTGTCCAAGTGAGATGTTTTCAATAC
TTAATTTCTGGAATTATGGAAATATTTGGGTATGCAGTGTTGATGTGTAAATACAACTCCTAGCCTAGATTGCTTGC
ATGCTATTTATGTTGATGTCGCGTGGGTATAGATATTTCTGGTATTATCGAATTACTTAATAAGATGAGGCCAATGA
GCTCCAATTCCGACGTCTAAGAAACCATTATTATTATGACATTAACCTATAAAAATAGGCGTATCACGAGGCCCTTC
CGTCTTCACCTCGAGTCCCTATCAGTGATAGAGATTGACATCCCTATCAGTGATAGAAATACTGAGCACATCAGCAG
GACGCACTGACCGGATCCAAAGGAGAGGGAAATGAATATCGCGGACATGAACGTGGCTGATATGAACGTTGAAA
ATGCAGCCAAAGTGCTGAAGGAGTTGGGGCACCCCACCCGCTTGGCATTATTTCGCCTTTTAGTTAAAGGGGGGTA
CACTGGCGTTGCAGTAGGGCAACTGCAGGAGGCTTTACAAATCCCTGGGTCCACTTTATCCCACCACATCTCGGCTT
TGATGTCTGCTGGTATCATCTCGCAGCGTCGTGAAGGCCGCGTACTGTATTGCGTCCCCGATTACGAGTTGTTACAG
GGTTTAGTACACTTCTTGCAGGACCAGTGTTGTAGCGGACAGTGAGGATCCTGC*TCTAGA*

arsP promoter (on complement), highlighted in pink
AA-promoter, highlighted in blue
arsR, highlighted in green
*Bam*HI sites are underlined, *Xba*I and *Eco*RI sites are in italics

Text S4. Assessment of Nd-biosensor activity on agar

A 10-µl drop of arsenical (250 µM As^{III} or 40 µM $MMAs^{III}$) was added at the centre of two ¼ TSB agar plates (containing 50 µg/ml kanamycin). After complete absorption on the agar, the Nd-biosensor strain was spread as a lawn on ¾ of the agar surface, and a positive control (Nd-biosensor-KO) was streaked on the leftover space. After overnight aerobic incubation at 37°C, the plates were imaged on a Fusion Fx (Vilber, Marne-la-Vallée, France) instrument. The fluorescence response observed was much stronger with $MMAs^{III}$ than with As^{III} (Fig. S5). The same experiment was attempted with the Sp-biosensor strain but yielded insufficient signal.

Text S5. Whole-genome sequencing of selected isolates

gDNA was extracted with the Monarch Genomic DNA Purification Kit (NEB, Ipswich, MA, USA) and assessed for quantity, quality and purity using a Qubit 4.0 fluorometer (Qubit dsDNA HS Assay kit; Q32851, Thermo Fisher Scientific, Waltham, MA, USA), an Advanced Analytical FEMTO Pulse instrument (Genomic DNA 165 kb Kit; FP-1002-0275, Agilent Technologies, Santa Clara, CA, USA) and a DS-11 UV-Vis spectrophotometer (Denovix, Wilmongton, DE, USA), respectively.

Multiplexed SMRTbell libraries were prepared for sequencing on the Revio exactly according to the PacBio guideline entitled: "Preparing multiplexed whole genome and amplicon libraries using the HiFi plex prep kit 96 " – Part Number 103-418-800 REV03 (DEC 2024). The only deviation made to this procedure and checklist document, was that 300-1000 ng of gDNA in 100 µL was used to shear the gDNA using a Covaris g-TUBE (Covaris, Woburn, MA, USA) following the PacBio technical note for Covaris g-TUBE DNA shearing for SMRTbell prep kit 3.0. Briefly, thereafter the sheared gDNA was concentrated and cleaned using 1 x SMRTbell clean-up beads. The samples were then quantified and qualified to be in the range of 7-12Kb using a Qubit 4.0 fluorometer (Qubit dsDNA HS Assay kit; Q32851, Thermo Fisher Scientific, Waltham, MA, USA) and an Advanced Analytical FEMTO Pulse instrument (Genomic DNA 165 kb Kit; FP-1002-0275, Agilent Technologies, Santa Clara, CA, USA), respectively. The rest of the procedure as referenced above was followed including end-repair & A-tailing, ligation of barcoded overhang adapters and then purification of the library using AMPure PB beads as well as a nuclease treatment. The libraries were pooled with other microbial libraries and purified using AMPure PB beads (3Kb size selection). Library pool concentration and size was again assessed using a Thermo Fisher Scientific Qubit 4.0 fluorometer and an Advanced Analytical FEMTO Pulse instrument (as described above), respectively. Instructions in SMRT Link Sample Setup were followed to prepare the SMRTbell library for sequencing (PacBio SMRT Link v13.1). Shortly, using components from a Revio polymerase kit + cleanup beads bundle (PacBio, PN 102-817-600), the PacBio standard sequencing primer was annealed to the SMRTbell libraries, next the Revio DNA Polymerase was bound, and the polymerase bound complex was bead-based purified. Finally, the Revio sequencing control DNA was diluted and spiked into the complex prior to pipetting onto the thawed Revio sequencing plate (PacBio, PN102-587-400). The Revio deck was setup as directed from the SMRTLink software and included laying out tips, sequencing plates and Revio SMRT Cell trays containing 4 x SMRT cell 25M (PacBio, PN 102-202-200) into their designated locations. The libraries were generally loaded at an on-plate concentration of 135-330pM using adaptive loading. SMRT sequencing was performed on the Revio controlled by instrument software 13.0.0.212033 or 13.1.0.221972 and with a 30-h movie time. All steps post gDNA extraction were performed at the Next Generation Sequencing Platform, University of Bern, Switzerland.

Text S6. Bioinformatics analysis

High-fidelity (HiFi) metagenomic reads from isolates were assembled into consensus contigs using Flye (v2.9.3 and v2.9.5), yielding high-quality genomes. The completeness and contamination of the genomes were assessed using CheckM2 (v1.0.2), and taxonomic classification was performed with GTDB-Tk (v2.4.0) using the default database (v220). Functional annotation was conducted with Prokka (v1.14.6), incorporating a custom HMM database for arsenic metabolism (*ars*) genes from Keren et al. [1], extended to include *arsK* [2] and *arsW* [3]. Sequence alignments were performed in SnapGene (v8.0.2) using Clustal Omega (v1.2.4) or MUSCLE (v3.8.1551) with default parameters. The analyses were conducted on the EPFL

high-performance computing (HPC) cluster, utilizing *SLURM* (v23.11.10) and *Apptainer* (v1.2.5). Each node consisted of two Intel(R) Xeon(R) Platinum 8360Y processors running at 2.4 GHz, with 36 cores per processor (72 cores per node) and 3 TB of SSD storage.

Table S1. List of As chemical species and their abbreviations

**Chemical name**	**Abbreviation**
Arsenite	As^{III}
Arsenate	As^{V}
Inorganic arsenic (sum of As^{III} and As^{V})	iAs
Monomethylarsenite	$MMAs^{III}$
Monomethylarsenate	$MMAs^{V}$
Monomethylarsenic (valence state not specified)	MMAs
Dimethylarsenite	$DMAs^{III}$
Dimethylarsenate	$DMAs^{V}$
Dimethylarsenic (valence state not specified)	DMAs
Trimethylarsine	$TMAs^{III}$
Trimethylarsenic (valence state not specified)	TMAs

Supplementary figures

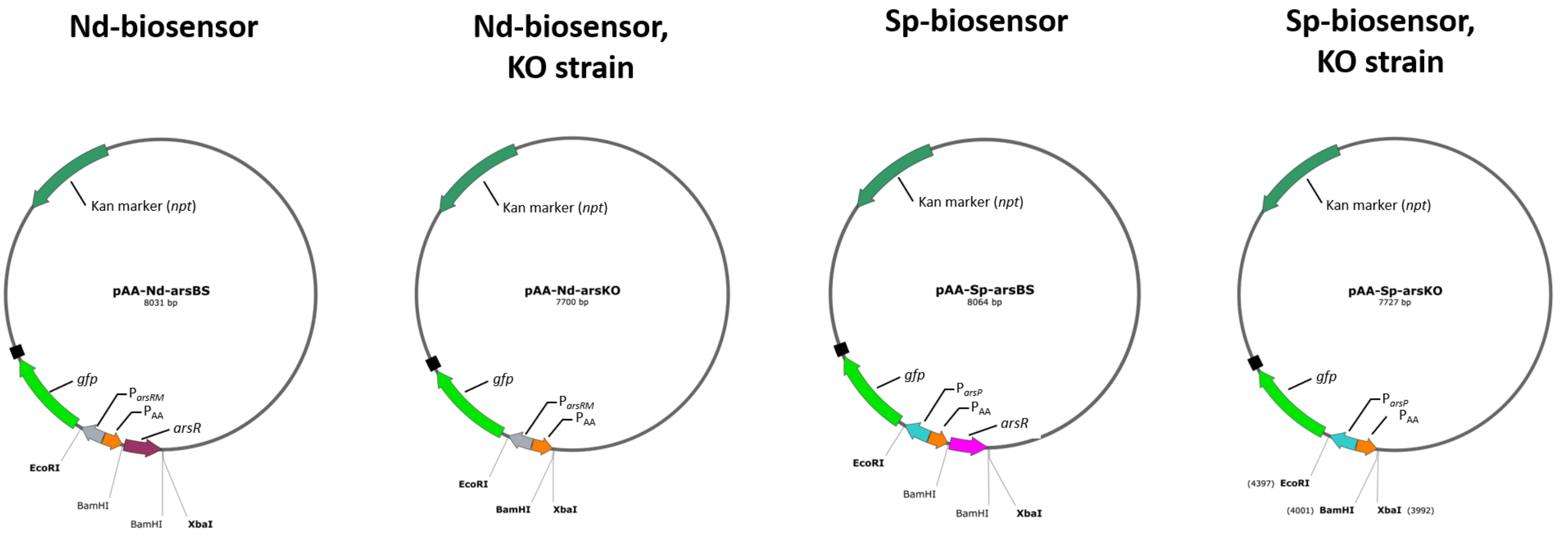

Figure S1: **Plasmids containing the $MMAs^{III}$ biosensor elements.** In plasmids pAA-Nd-arsBS and pAA-Nd-arsKO, *gfp* expression is controlled by the *arsRM* promoter from *N. denitrificans* (grey arrow). Expression of *gfp* in plasmids pAA-Sp-arsBS and pAA-Sp-arsKO is under control of the *arsP* promoter from *S. putrefaciens* (light blue arrow). *arsR* from *N. denitrificans* (purple arrow) and *S. putrefaciens* (pink arrow) are transcribed from a constitutive promoter (AA, orange arrow). The black square represents a lambda t0 terminator.

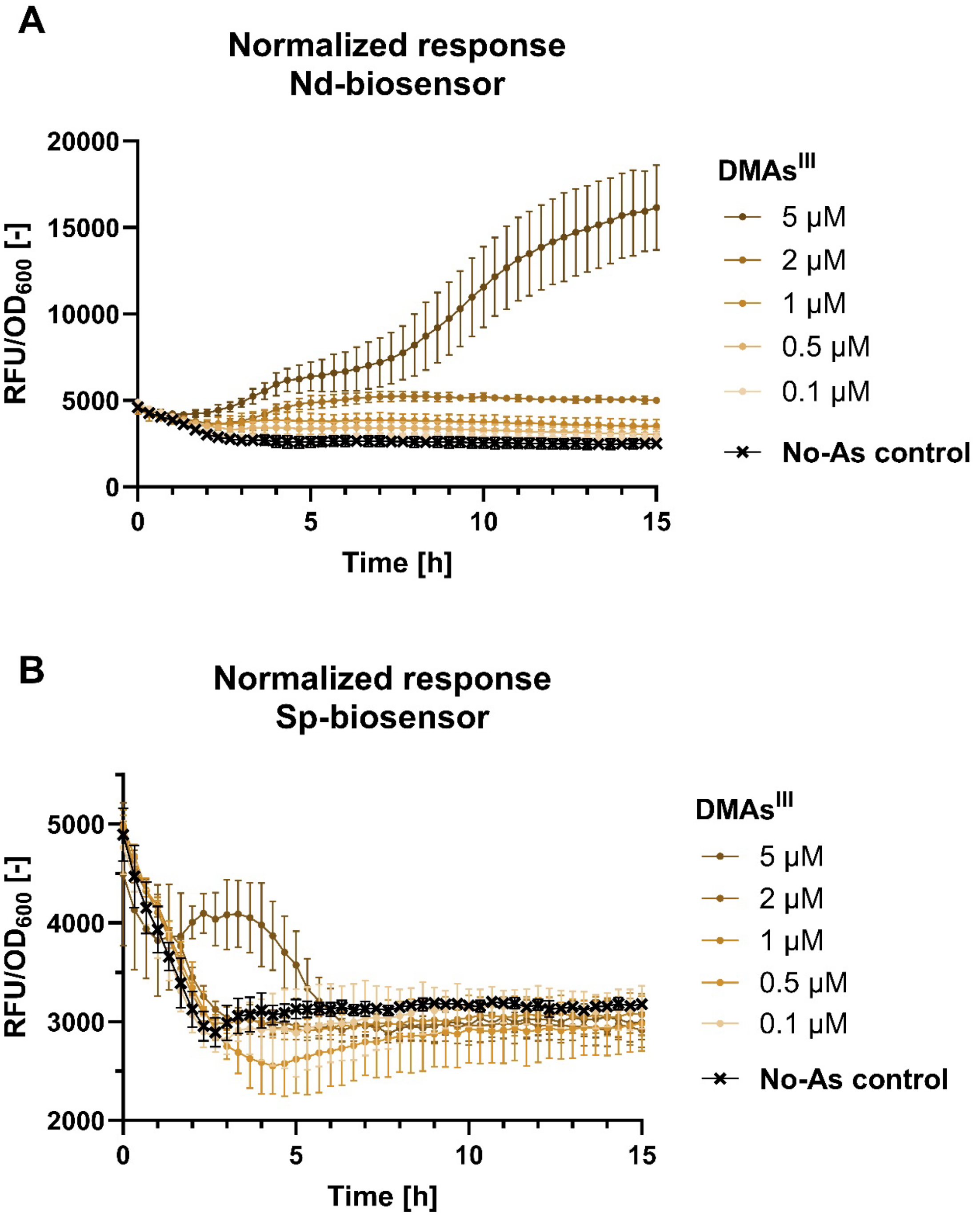

Figure S2: **Sensing of $DMAs^{III}$ by biosensor strains.** Normalized fluorescence response monitored in cultures of (A) Nd-biosensor and (B) Sp-biosensor amended with different concentrations of $DMAs^{III}$. Shown are means of four replicates, with error bars indicating standard deviation. RFU: relative fluorescence unit; OD_{600}: optical density read at 600-nm wavelength.

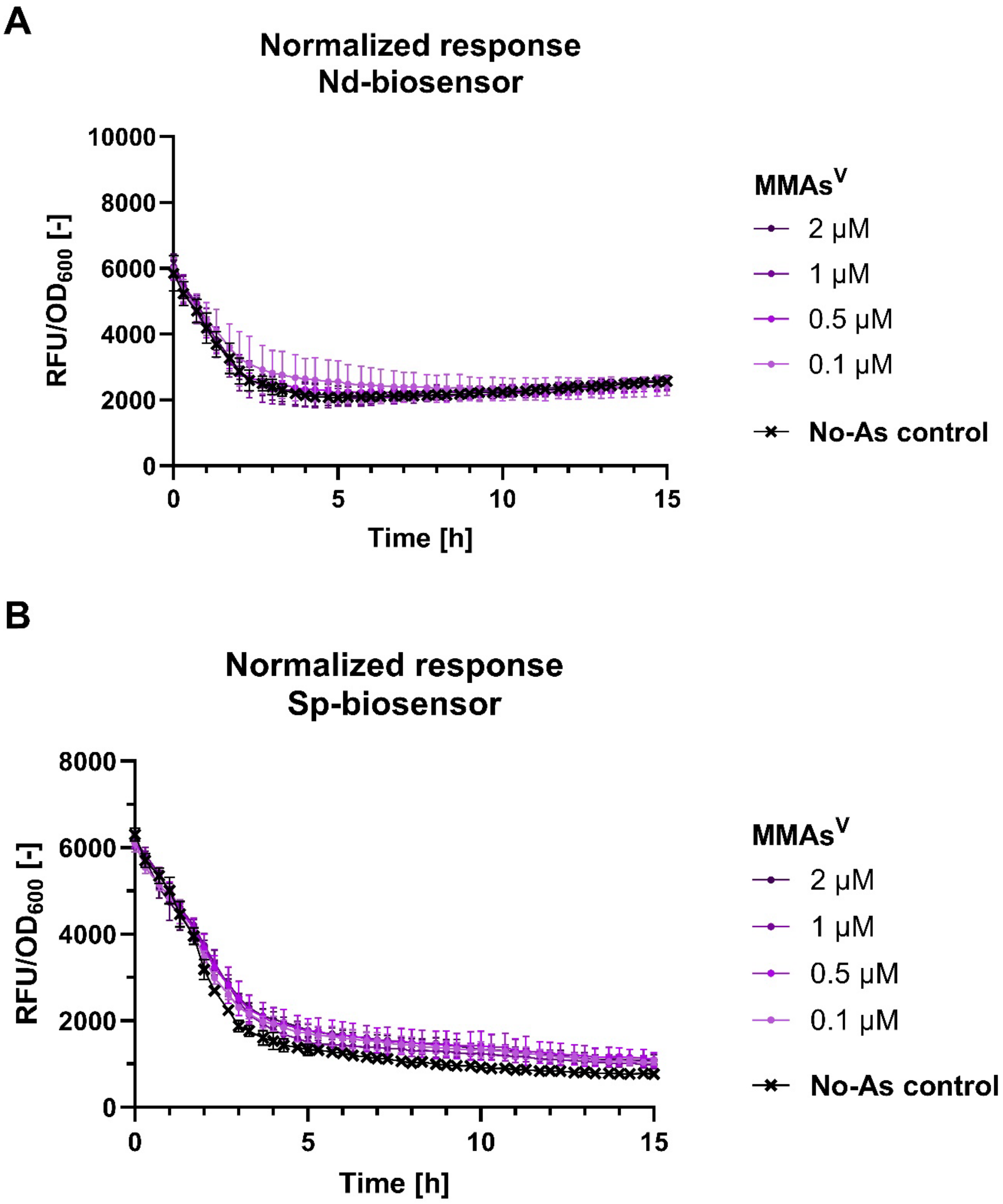

Figure S3: **Sensing of $MMAs^V$ by biosensor strains.** Normalized fluorescence response monitored in cultures of (A) Nd-biosensor and (B) Sp-biosensor amended with different concentrations of $MMAs^V$. Shown are means of four replicates, with error bars indicating standard deviation. RFU: relative fluorescence unit; OD_{600}: optical density read at 600-nm wavelength.

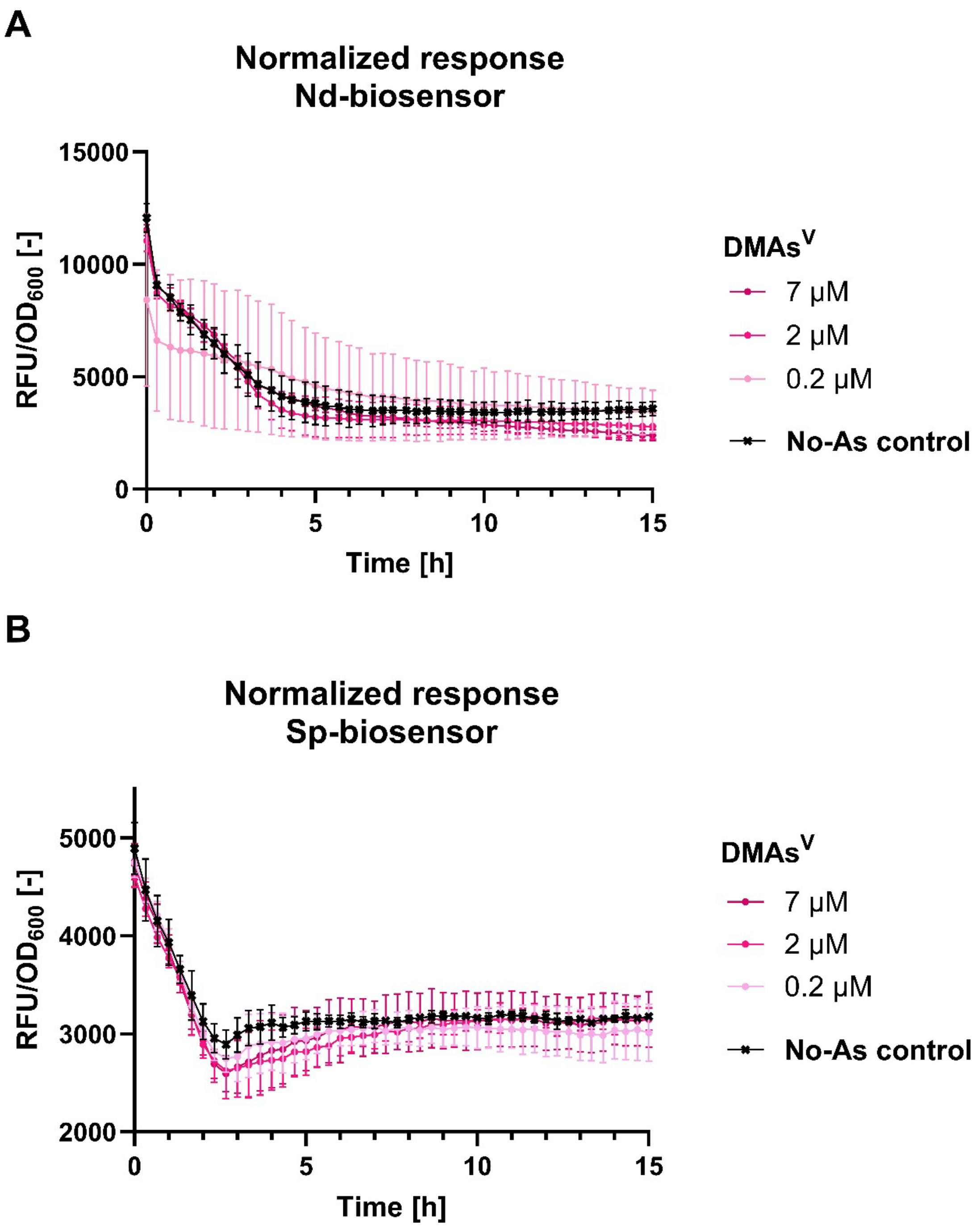

Figure S4: **Response of Nd-biosensor strain to DMAs^V.** Normalized fluorescence response monitored in cultures of Nd-biosensor amended with various amounts of DMAs^V. Shown are means of four replicates, with error bars indicating standard deviation. RFU: relative fluorescence unit; OD_{600}: optical density read at 600-nm wavelength.

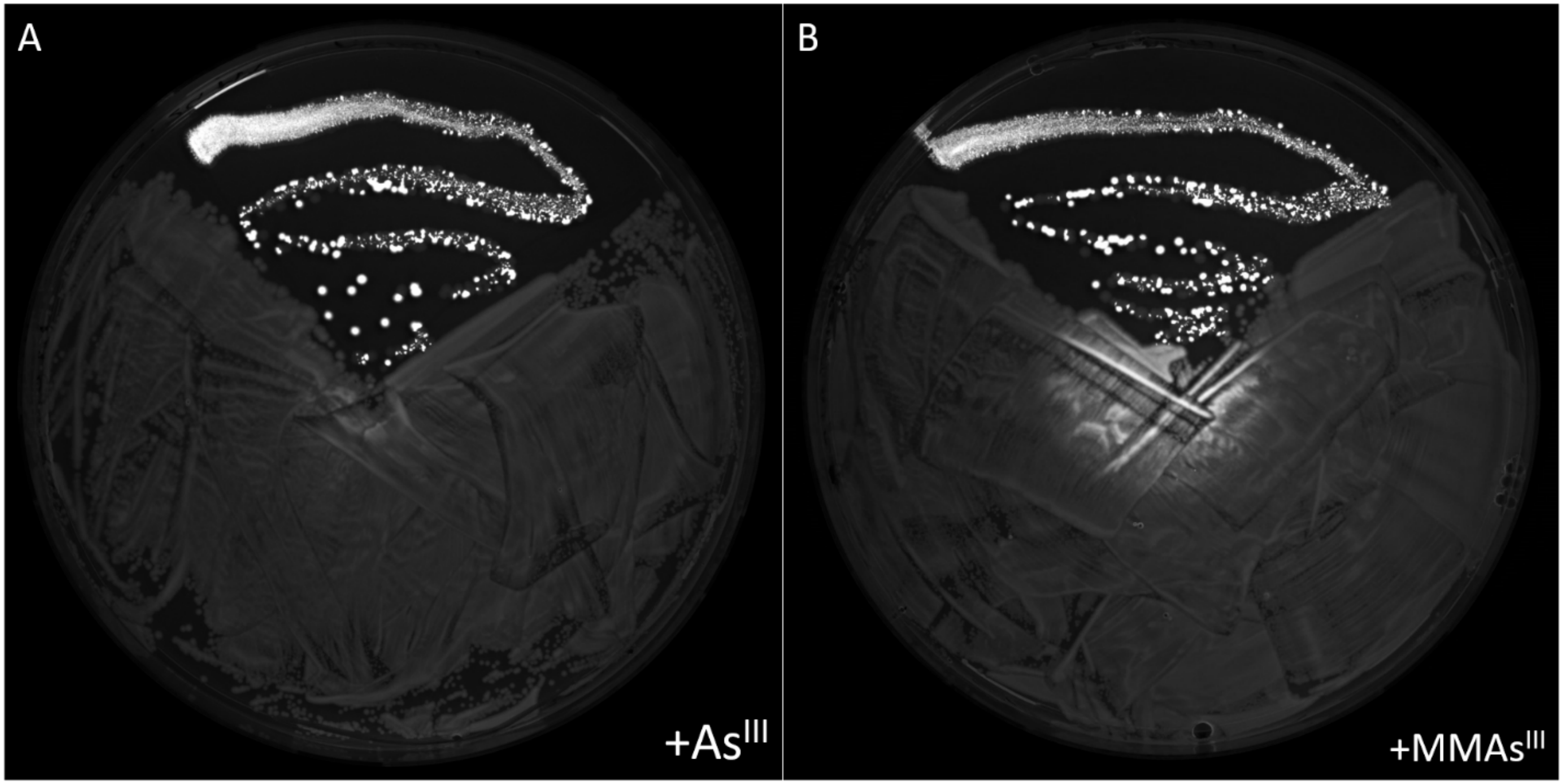

Figure S5: **Detection of As^{III} and MMAs^{III} by Nd-biosensor strain grown on agar.** A 10 µl-drop of As^{III} (A) or MMAs^{III} (B) was added at the centre of agar plates (¼ TSB with 50 µg/ml kanamycin). Nd-biosensor was then spread on ¾ of the agar surface, while Nd-biosensor-KO was streaked on the remaining quarter. The plates were incubated aerobically overnight at 37°C before fluorescence imaging. Arsenic concentrations in the drop: 250 µM and 40 µM for As^{III} and MMAs^{III}, respectively.

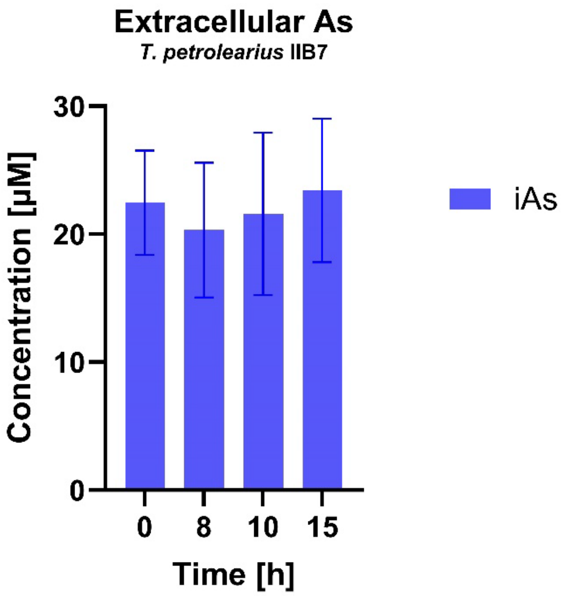

Figure S6: **Arsenic speciation dynamics in the culture medium of *T. petrolearius* IIB7.** iAs: inorganic arsenic.

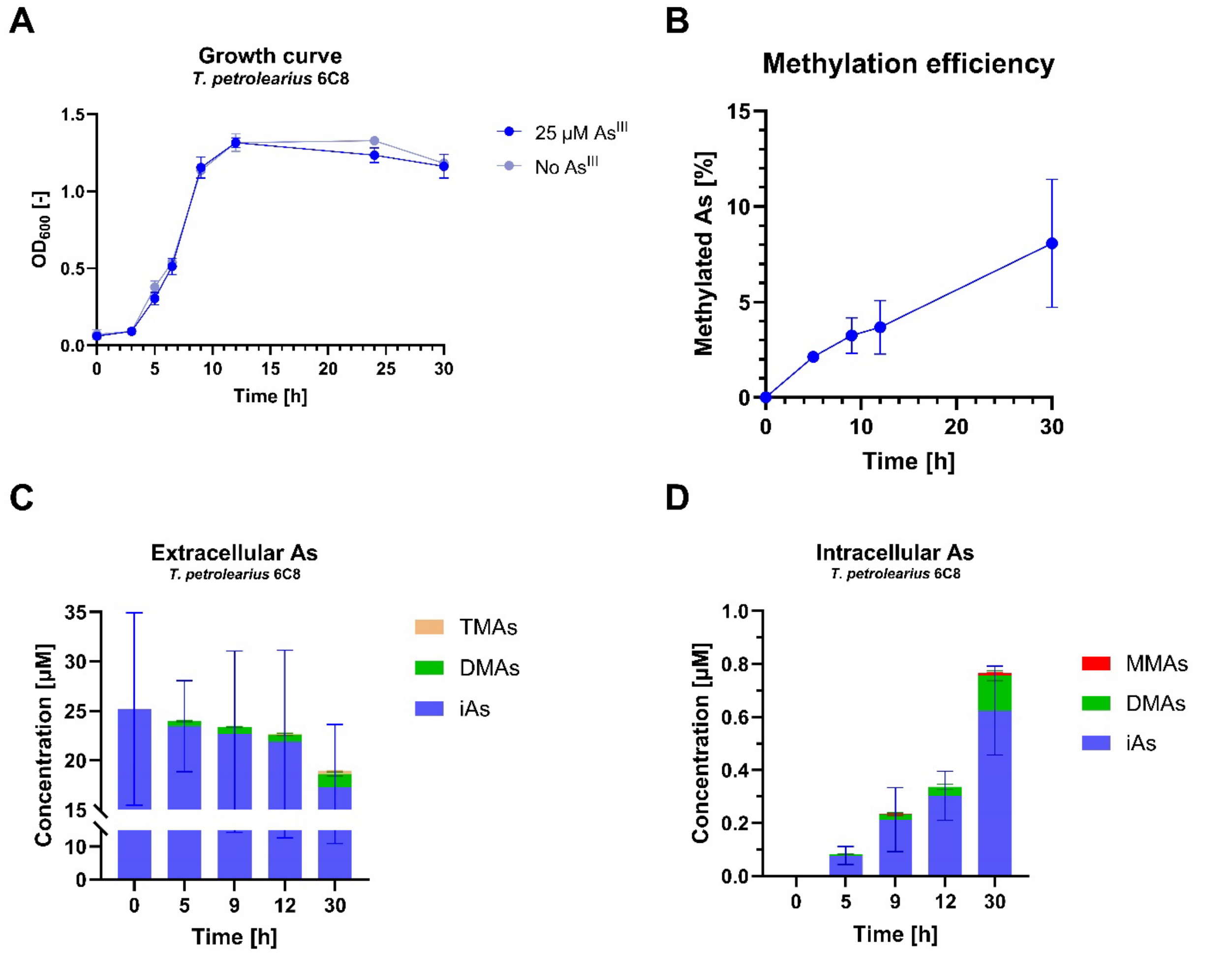

Figure S7: **As^{III} methylation dynamics in pure cultures of *T. petrolearius* 6C8.** (A) Growth profile and (B) methylation efficiency (proportion of methylated arsenic) in pure cultures of *T. petrolearius* 6C8. Arsenic speciation measured (C) in the culture medium and (D) inside the cells.

T. petrolearius 6C8

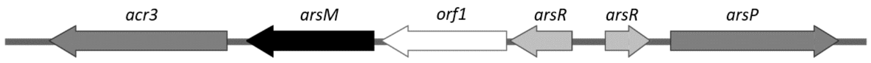

<u>Figure S8</u>: **Diagram of the *ars* operon in *T. petrolearius* 6C8.** A description of the genes and their product is as follows. *arsM*: As^{III} *S*-adenosylmethionine methyltransferase (ArsM), *orf1*: putative As^{V} reductase (Orf1), *acr3*: As^{III} permease (Acr3), *arsR*: arsenic-responsive transcriptional repressor (ArsR), *arsP*: putative $MMAs^{III}$ permease (ArsP).

References

1. Keren R, Méheust R, Santini JM *et al.* Global genomic analysis of microbial biotransformation of arsenic highlights the importance of arsenic methylation in environmental and human microbiomes. *Comput Struct Biotechnol J* 2022;**20**:559.

2. Shi K, Li C, Rensing C *et al.* Efflux Transporter ArsK Is Responsible for Bacterial Resistance to Arsenite, Antimonite, Trivalent Roxarsone, and Methylarsenite. *Appl Environ Microbiol* 2018;**84**, DOI: 10.1128/AEM.01842-18.

3. Chen J, Zhang J, Wu YF *et al.* ArsV and ArsW provide synergistic resistance to the antibiotic methylarsenite. *Environ Microbiol* 2021;**23**:7550–62.
